# Trafficking first, inflammation next: Epstein–Barr virus B cells seed central nervous system T-cell clusters

**DOI:** 10.1038/s41392-025-02470-x

**Published:** 2025-11-12

**Authors:** Green Kim, Jung Joo Hong

**Affiliations:** 1 National Primate Research Center, Korea Research Institute of Bioscience and Biotechnology (KRIBB), Cheongju, Chungcheongbuk Republic of Korea; 2 KRIBB School of Bioscience, Korea University of Science & Technology (UST), Daejeon, Republic of Korea

**Keywords:** Neuroimmunology, Infection

In a recent study published in *Nature*, Läderach et al. showed that Epstein–Barr virus (EBV) infection expands a T-bet+CXCR3 + B-cell subset that homes to the central nervous system (CNS) and organizes perivascular aggregates of activated T cells. This finding offers a mechanistic bridge between EBV infection and multiple sclerosis (MS) and suggests interference with chemokine-guided B-cell positioning, alongside B-cell depletion, as a therapeutic strategy to blunt neuroinflammation.^1,2^

The dynamic reprogramming of B cells is a feature of persistent viral infection and autoimmunity. EBV reshapes the B-cell fate via latent gene programs and epigenetic rewiring, generating proliferative, antigen-presenting states with tissue-homing potential. Age‑associated B cells (ABCs; also termed atypical memory B cells) accumulate during chronic infection and autoimmunity, are frequently T‑bet⁺CXCR3⁺, and follow CXCL9/10/11 gradients.^3^ Although epidemiology and serology strongly link EBV infection to MS, the route by which infected B cells gain CNS access and recruit pathogenic T cells remains unresolved.^3,4^

To address this gap, the authors used humanized mice carrying the principal MS-risk allele HLA-DRB1*15:01 and combined flow cytometry, ChipCytometry, single-cell transcriptomics with paired B-cell receptor sequencing, and light-sheet imaging. EBV infection expanded oligoclonal T-bet+CXCR3 + B cells in the blood, spleen, and brain. Shared B-cell receptor clones across compartments indicated directed trafficking rather than de novo local expansion. Three-dimensional imaging of cleared brains revealed dense human CD45+ and CD3+ immune aggregates along the meningeal and perivascular spaces, providing anatomical support for EBV-imprinted B-cell entry into the CNS (Fig. 1).^1^

**Fig. 1:**
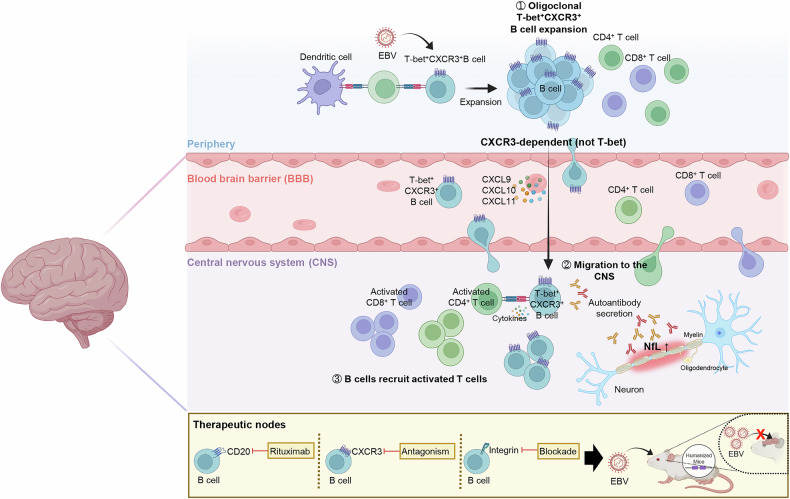
EBV-imprinted B-cell trafficking and T-cell recruitment to the CNS. EBV expands T-bet+CXCR3 + B cells in the periphery. These cells home in on the CNS along CXCL9/10/11 gradients, lodge in perivascular and meningeal niches, present antigen and cytokines, and recruit activated CD8+ and CD4 + T cells. Therapeutic nodes include anti-CD20 depletion, CXCR3 antagonism, and integrin blockade. Combination approaches restrict brain infiltration in humanized mice. This figure was created with BioRender.com. BBB blood–brain barrier, CNS central nervous system, EBV Epstein–Barr virus, NfL neurofilament light chain

Next, the authors tested whether EBV directly imprints this promigratory state. In vitro infection of sorted human B cells rapidly induced T- bet and favored the outgrowth of CXCR3-positive lymphoblastoid cell lines. In the CNS of the infected mice, T-bet+CXCR3 + B cells localized to submeningeal/perivascular niches and recruit T cells via B-cell-derived CXCR3-ligand chemokines (CXCL9/10), together with CCL3/CCL4/CCL5. Supernatants from EBV-transformed B cells, which contain CXCL9/10 and CCL3/CCL4/CCL5 along with TNF, drove migration of activated CD4+ and, to a lesser degree, CD8 + T cells; neutralizing these chemokines or pharmacological CXCR3 antagonism reduced migration.^1^

Given the clinical efficacy of anti-CD20 therapy in relapsing MS but its limitations in progressive disease, this study evaluated whether trafficking blockade augments depletion. In humanized mice, rituximab or a CXCR3 inhibitor reduced leukocyte entry into the brain, and the combination produced the largest effect. Integrin blockade (natalizumab) also curtailed B-cell brain homing. These results suggest that the CXCR3 axis is a tractable upstream node that complements B-cell depletion and integrin adhesion blockade.^2^

In the aforementioned *Nature* study by Läderach et al., the identity of the neuroinvasive B cells was defined: Single-cell analyses showed increased expression of the adhesion molecules ALCAM and LFA-1, increased antigen-presentation programs, reduced CXCR5 expression, and oligoclonal expansion. EBV transcripts were abundant in clonally related cycling and plasmablast populations, whereas the T-bet+CXCR3+ memory subset carried few viral messages, consistent with latency and immune evasion. Importantly, CNS entry correlated with CXCR3, not T-bet, expression on B cells, making CXCR3 the dominant determinant of neuroinvasion.^1^

While prior studies have implicated EBV-reactive and autoreactive B-cell responses in MS, the present results suggest that the initiating step is positional: EBV-imprinted antigen-presenting B cells are guided to CNS borders where they recruit and license inflammatory T cells and amplify tissue damage. Collectively, the study identifies T-bet+CXCR3 + B cells as effectors that connect a ubiquitous latent virus to focal neuroinflammation and elevates chemokine-guided positioning to the level of a druggable axis.^5^

Methodologically, these conclusions are strengthened by convergence across modalities and functional perturbations in vivo. Nonetheless, key unknowns remain, and define a translational agenda. What CNS-derived cues establish CXCR3 ligand gradients? Which antigens are recognized by the dominant B-cell clones that enter the brain, and do these clones differ across MS stages? Can EBV-specific vaccination or adoptive T-cell therapy eliminate upstream drivers without collateral toxicity? Answering these questions will clarify how to combine anti-CD20, CXCR3 antagonists, integrin blockade, and EBV-directed strategies for durable control of neuroinflammation.

## Data Availability

No new data were generated or analyzed in this Research Highlight. All findings discussed are derived from and supported by the cited literature.
